# Characterization of oogonia stem cells in mice by Fragilis

**DOI:** 10.1007/s13238-019-00654-0

**Published:** 2019-09-26

**Authors:** Xiaoyan Sheng, Chenglei Tian, Linlin Liu, Lingling Wang, Xiaoying Ye, Jie Li, Ming Zeng, Lin Liu

**Affiliations:** 1 State Key Laboratory of Medicinal Chemical Biology, College of Life Sciences, Nankai University, Tianjin, 300071 China; 2 Department of Cell Biology and Genetics, College of Life Sciences, Nankai University, Tianjin, 300071 China

**Dear Editor,**

Identification of oogonia stem cells would have great potentials in infertility treatment and fertility preservation. Here we tested whether Fragilis/Ifitm3 can identify oogonia stem cells in fetal mouse ovaries and their molecular features, if it can, and whether the oogonia stem cells marked by Fragilis can be found in the postnatal ovaries. It is well established that the primordial germ cells (PGCs) in embryonic gonads undergo multiple cell divisions during migration to the genital ridge to produce oogonia that can differentiate into meiotic cells (Saitou and Miyauchi, 2016), and thus are definitive oogonia stem cells. Moreover, primordial germ cells-like cells (PGCLCs) induced from ESCs or iPSCs that resemble PGCs *in vivo* also are able to form functional oocytes that produce live pups (Hayashi et al., 2012; Hikabe et al., 2016). We essentially investigated the utility of Fragilis as a germ cell marker during different developmental stages. Our analysis suggests that Fragilis expression on cell surface might be a useful PGC marker at fetal E10.5 and E12.5, but its expression decreases thereafter and notably Fragilis is predominantly expressed in somatic stroma or theca and epithelial cells postnatally. Moreover, the Fragilis-expressing cells in fetal gonads are competent to undergo meiosis and generate functional oocytes in a reconstituted ovary assay, but those from postnatal gonads are not similarly developmentally competent. Comparison of the fetal and postnatal Fragilis^+^ cells in molecular signatures and function reveals that Fragilis expression at cell surface can specifically identify oogonia stem cells in fetal gonads, but its expression does not detect oogonia stem cells in postnatal ovaries.

PGCs highly express specific germ cell marker genes, notably *Stella*/*Dppa3*, *Blimp1*/*Prdm1*, *Ddx4*/*Vasa*/MVH, *Oct4* and *Fragilis*/*Ifitm3* (Noce et al., 2001; Saitou et al., 2002; Tanaka et al., 2004; Ohinata et al., 2005; Okamura et al., 2008; Sabour et al., 2011). These germ cell specifiers also are conserved in humans (Kobayashi and Surani, 2018). Fragilis, as transmembrane protein, could be potentially useful for identification and sorting of PGCs or oogonia stem cells. Hence, we systematically examined the molecular signatures of Fragilis-sorted cells from fetal ovaries, and also compared with those of postnatal ovaries. We explored the expression pattern of Fragilis by co-immunostaining with known germ cell markers Vasa (Ddx4) or Dazl, in mouse fetal ovaries from E10.5, E12.5, E13.5 to E16.5, and compared with the postnatal ovaries from one and six-week old mice by immunofluorescence microscopy. Fragilis was specifically expressed at the cell surface, and Vasa and Dazl were mainly localized to the cytoplasm of germ cells as reported (Figs. 1A and S1).

**Figure 1:**
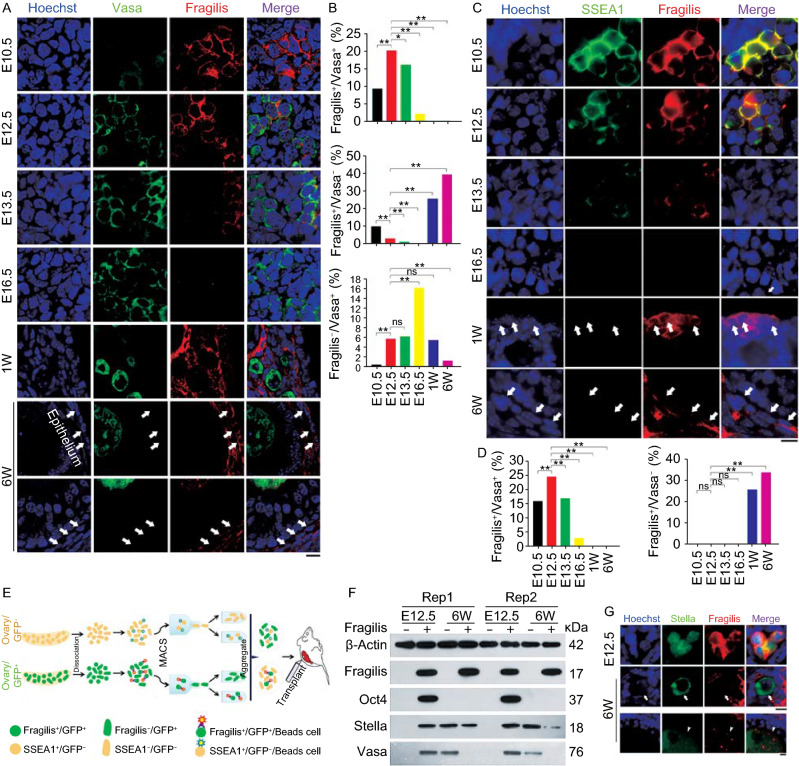
**Expression pattern of Fragilis in mouse fetal and postnatal ovaries**. (A) Representative confocal images showing co-immunostaining of Vasa (green) with Fragilis (red) in sections of E10.5, E12.5, E13.5 and E16.5 or one week (W) and 6-week old mouse ovaries. White arrows indicate Fragilis^+^/Vasa^−^ cells in the epithelia, stromal or theca cells. Scale bar = 10 μm. (B) Proportion (%) of Fragilis^+^/Vasa^+^ cells, Fragilis^+^/Vasa^−^ cells, and Fragilis^−^/Vasa^+^ cells in mouse ovaries. X^2^ test (*n* ≥ 600 cells counted from three samples for each group). *P < 0.05, **P < 0.01, compared with E12.5 gonad. ns, non-significant difference. (C) Co-immunostaining by epi-fluorescence of SSEA1 (green) with Fragilis (red) in E10.5, E12.5, E13.5 and E16.5, or one week (W) and 6-week old mouse ovaries. White arrows indicate Fragilis^+^/SSEA1^−^ cells in the epithelia, stromal or theca cells. Scale bar = 10 μm. (D) Proportion (%) of Fragilis^+^/SSEA1^+^ cells, and Fragilis^+^/SSEA1^−^ cells in mouse ovaries. X^2^ test (*n* ≥ 600 cells counted). *P < 0.05, **P < 0.01, compared with E12.5 ovaries. Three repeats. (E and F) Isolation and characterization of Fragilis^+^ cells sorted from mouse ovaries. (E) Overview of the protocol for isolation of Fragilis^+^ cells and experimental design for transplantation. (F) Protein expression levels by Western blot of Fragilis, Oct4, Stella and Vasa in Fragilis^+^ cells and Fragilis^−^ cells from E12.5 and 6-week mouse ovaries. β-Actin served as loading control. (G) Co-immunofluorescence of Stella (green) and Fragilis (red) in E12.5 and 6-week old mouse ovaries. Stella mainly in nuclei and Fragilis on the membrane are co-expressed in E12.5 ovaries. In 6-week mouse ovaries, Stella is localized in the cytoplasm of oocytes. Most of Stella positive oocytes are Fragilis negative (white arrows), and few are also positive for Fragilis in which Fragilis is expressed in the cytoplasm of oocytes of secondary and mature/antral follicles (white arrowheads). Scale bar = 10 μm

During germ cell development in the fetal ovary, while Fragilis fluorescence signal intensity decreased from E12.5 to E16.5, the fluorescence intensity and number of Vasa and Dazl gradually increased (Figs. 1A and S1). Proportion of germ cells expressing Fragilis increased at E12.5 compared to E10.5 and remarkably decreased at E13.5 when germ cells enter meiosis, and very rare cells expressed Fragilis at E16.5 compared to other periods (Figs. 1A and S1B). Thus, PGCs at E10.5 and E12.5 are featured with co-expression of membrane Fragilis and cytoplasmic Vasa or Dazl. In addition, percentage of cells expressing only Fragilis but without Vasa also decreased during fetal development.

Fragilis^+^ cells also can be found in the postnatal ovaries, and Fragilis^+^/Vasa^−^ or Dazl^−^ cells mostly were visible in the cortex and ovarian surface epithelium (OSE) (white arrows) (Figs. 1A and S1). Careful counting of the fluorescent cells showed that PGCs simultaneously positive for both membrane Fragilis and cytoplasmic Vasa were mostly seen at E12.5 and could not be found in postnatal ovaries (Fig. 1B). In contrast, Fragilis^+^ but Vasa^–^ cells were few in fetal E12.5, and became significantly increased in postnatal ovaries. Vasa^+^ but Fragilis^−^ cells can be found in low frequency in both fetal E12.5 and postnatal ovaries (Fig. 1B). However, expression of Vasa and Fragilis can also be found in the cytoplasm of developing oocytes (Fig. S1A). Thus, the expression pattern of Fragilis, Vasa and Dazl in the postnatal ovary differs remarkably from that of the fetal ovary. This prompted us to further examine the properties of the Fragilis^+^ cells.

Expression of SSEA1 in membrane and nuclear expression of Oct4 serve as important markers to identify PGCs and pluripotent stem cells (Hayashi et al., 2012; Guo et al., 2017). Expression and cellular localization of SSEA1 and Fragilis were similar in fetal ovaries. SSEA1 and Fragilis were co-expressed on the cell surface (Fig. 1C), consistent with SSEA1 being used for sorting PGCLCs differentiated from pluripotent stem cells (Hayashi et al., 2012). Fragilis^+^/SSEA1^+^ PGCs exhibited highest frequency at E12.5 and the frequency was reduced after meiosis entry and became few at E16.5 (Fig. 1D). Oct4 was expressed in the nuclei of PGCs with membrane Fragilis (Fig. S2A). Fragilis^+^ cells with nuclear Oct4 expression were abundant in the fetal E12.5 ovary and reduced after E13.5 (Fig. S2A). Co-localized expression pattern of SSEA1 and Vasa corroborated with that of Fragilis and Vasa (Fig. S2B). By E16.5 when most meiocytes reached pachytene stage, they almost completely lost expression of SSEA1 and Oct4, like that of Fragilis (Figs. 1C, 1B and S2A).

In postnatal ovaries, cells positive for Fragilis can be found in the cortex and OSE but these cells were negative for SSEA1 and Oct4 (Figs. 1, S2 and S6). To further characterize Fragilis positive cells in postnatal ovaries, we performed co-immunostaining with Fragilis to reveal expression of Vasa, the proliferative marker PCNA, and nuclear expression of Gata4, crucial protein in granulosa cell development and function (Bennett et al., 2013), or Foxl2, a specific marker for granulosa cells (Pisarska et al., 2011). In E12.5 ovaries, PGCs co-expressing nuclear PCNA and membrane Fragilis suggest their proliferative capacity (white arrows), but somatic or granulosa cells that lacked Fragilis were also positive for PCNA (arrowhead) (Fig. S3A). In 6-week mouse ovaries, some Fragilis positive cells also showed proliferative capacity (white arrows), while others did not (arrowhead).

Fragilis^+^ cells lacked expression of Gata4 in E12.5 ovaries (white arrow), and Gata4 was exclusively expressed in the nuclei of Fragilis^−^ cells (arrowhead), which are likely progenitor cells for granulosa cells (Fig. S3B). By contrast, in 6-week old mouse ovaries, many Fragilis^+^ cells expressed nuclear Gata4 (white arrow) (Fig. S3B) and were incompatible with nuclear expression of Foxl2 (Fig. S3C). Moreover, cells expressing Fragilis in the postnatal ovaries were observed at different locations and varied in size and shape. Cells expressing Fragilis could be seen in three types, epithelial cells, stroma cells and growing germinal vesicle (GV) oocytes (Fig. S3D and S3E). Compared to size and round-shape of Fragilis positive cells from E12.5 ovaries, Fragilis positive cells at epithelium and stroma in the postnatal ovaries appeared smaller and flattened.

We further characterized Fragilis-sorted cells isolated from fetal and postnatal ovaries by magnetic activated cell sorting (MACS). Re-aggregation with E12.5 fetal somatic cells of the sorted cells to reconstitute ovaries was followed by transplantation under kidney capsules of bilaterally ovariectomized female recipient mice (Fig. 1E). To distinguish Fragilis^+^ cells from somatic SSEA1^−^ cells in the reconstituted ovaries (rOvaries), we sorted Fragilis^+^ cells from actin-GFP mice, and SSEA1^−^ cells from wild-type mice. Western blot analysis showed that the sorted cells were highly pure in that Fragilis^−^ cells did not express Fragilis protein (Figs. 1F and S4A). Consistently, Fragilis^+^ cells from E12.5 ovary expressed Oct4, Stella and Vasa, in addition to Fragilis, but Fragilis^−^ cells did not express these proteins. Unlike those of fetal ovaries, Fragilis^+^ cells from 6-week ovaries did not express Oct4 and Vasa, and yet Fragilis^−^ cells expressed Vasa, in agreement with the above immunofluorescence data (Figs. 1A and S1A).

Notably, important germ cell marker protein Stella/Dppa3 was expressed in both Fragilis^+^ and Fragilis^−^ cells in the adult ovaries (Fig. 1F). Immunofluorescence co-staining of Stella and Fragilis revealed nuclear Stella expression and membrane Fragilis in the same germ cells in fetal E10.5 and E12.5 ovaries, but translocation of Stella to cytoplasmic expression with developmental progression despite unclear mechanisms yet (Figs. 1G and S5). Stella was diffusely stained in the cytoplasm of oocytes with or without Fragilis in postnatal ovaries (Fig. 1G). In addition to general expression of Fragilis at cell surface of stromal and theca cells, Fragilis residue also can be found in the cytoplasm of GV oocytes within secondary, growing and mature/antral follicles in adult mice (Figs. 1G, S5 and S6A). Oocytes isolated from growing and mature/antral follicles of adult mice validated expression of Fragilis in their cytoplasm (Fig. S6B). Ovaries from adult mice expressed Fragilis protein level slightly higher that that of E12.5 gonad (Fig. S6C). SSEA1^+^ cells expressed Fragilis, Oct4 and Stella proteins, in contrast to SSEA1^−^ cells (Fig. S4B), also consistent with immunofluorescence, showing co-localized Fragilis and membrane SSEA1 or nuclear Oct4 in E12.5 ovaries but not in postnatal ovaries (Figs. 1C and S6D). These results further indicate that Fragilis^+^ cells differ between E12.5 and adult ovaries. Fragilis^+^ cells in E12.5 ovaries manifest properties of oogonia stem cells, but those cells in adult ovaries do not.

The most critical criteria or gold standard to evaluate oogonia stem cells is their differentiation ability by determining whether they are able to undergo normal meiosis (Handel et al., 2014). We analyzed homologous chromosome pairing and synapsis by immunofluorescence of SCP3/SCP1 elements and recombination by MLH1, key events taken place at meiosis prophase I (Handel et al., 2014). The synchronized interactions between oogonia and gonadal somatic cells are important to ensure meiosis entry and normal folliculogenesis. Indeed, E12.5 PGCs aggregated with somatic cells including pre-granulosa cells at the same developmental stage form oocytes following transplantation into kidney capsule and these oocytes are fertilizable and produce live young (Shen et al., 2006). Fragilis^+^ cells isolated from E12.5 or 6-week ovaries were aggregated with SSEA1^−^ somatic cells of E12.5 gonad, and treated with 3 µmol/L retinoic acid for 24 h to enrich meiotic cells, followed by transplantation into kidney capsules of bilaterally ovariectomized recipient mice for 6 days. Fragilis^+^ cells from fetal E12.5 ovaries underwent normal meiosis as evidenced by proper homologous pairing revealed by co-immunostaining of SCP3/SCP1, like meiocytes of E16.5–E17.5 female gonad (Figs. 2A, S7A and S7B). The number of SCP elements and proportion of correct number of homologues (20) of these cells formed in the aggregates were comparable to those of E17.5 ovaries (Fig. 2A). Moreover, these paired homologues underwent meiotic recombination as shown by MLH1 foci spotted on SCP3 lateral elements. The average number of MLH1 foci (~25) per spread was also similar to that of E17.5 ovaries (Fig. 2B). Hence, Fragilis^+^ cells from fetal E12.5 gonad underwent normal meiosis. Under the same experimental conditions, however, we were unable to find meiocytes that showed SCP3/SCP1 elements for homologous pairing in the spread or sections from Fragilis^+^ cells isolated from 6-week old ovaries (Figs. 2F and S7B).

**Figure 2:**
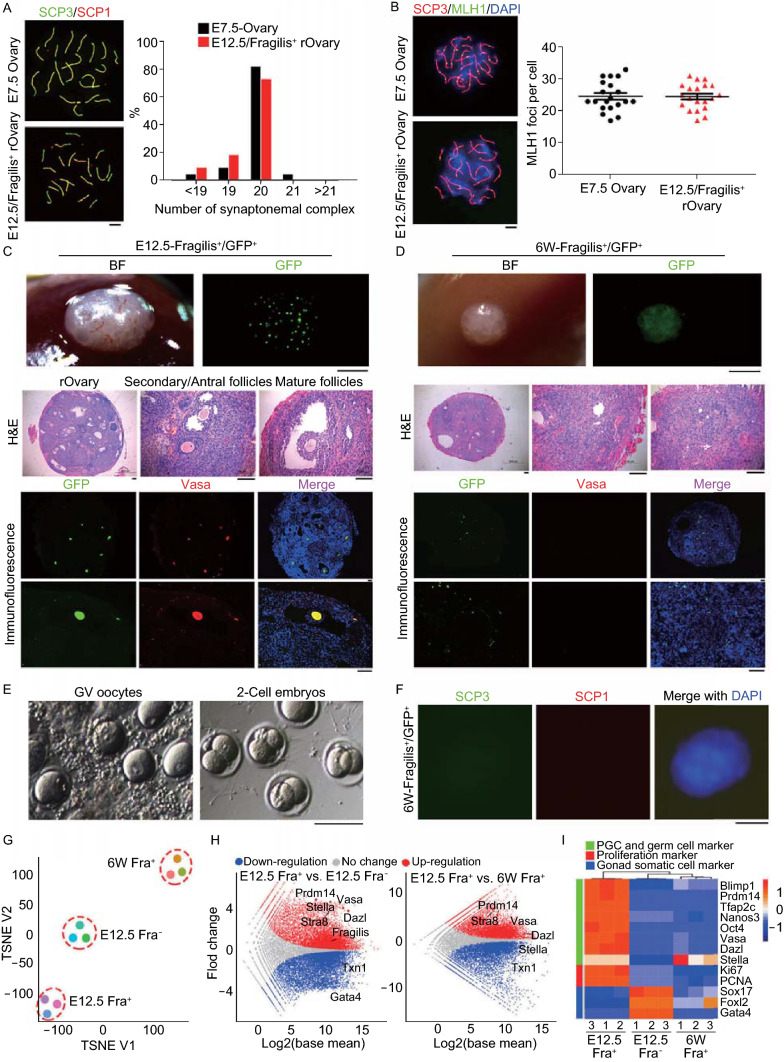
**Neo-meiosis and functional test of oocytes developed from Fragilis**^**+**^**cells**. (A) Immunofluorescence of SCP1 (red) and SCP3 (green, appear as yellowish by merge with SCP1) showing pachytene spread of E12.5 Fragilis^+^ cells obtained from aggregates with fetal E12.5 somatic cells 6 days following transplantation, compared with those of E17.5 ovaries. Right panel, Percentage of synaptonemal complex elements based on count of spread at pachytene (*n* = 22). Scale bar = 5 μm. (B) Representative immunofluorescence of MLH1 foci at pachytene stage by co-immunostaining of SCP3 (red) and MLH1 (green). Right panel, Statistics of MLH1 foci represents 20 spread per each group. Scale bar = 5 μm. (C) Morphology of reconstituted ovaries 28 days following transplantation of E12.5 Fragilis^+^ cells aggregated with E12.5 gonadal somatic cells into kidney capsules of ovariectomized mice (*n* = 8). Scale bar = 1 mm (upper panel). Middle panel, Follicles are shown in sections by H&E staining. Scale bar = 100 μm. Bottom panel, Co-immunostaining of GFP (green) and Vasa (red) in reconstituted ovaries. BF, bright-field. Nuclei were stained in blue by Hoechst. Scale bar = 100 μm. (D) Morphology of grafts 28 days following kidney capsule transplantation of 6-week Fragilis^+^ cells aggregated with E12.5 gonadal somatic cells (*n* = 3). No follicles but GFP^+^ somatic cells are seen. (E) Morphology of GV oocytes isolated from reconstituted ovaries developed from E12.5 Fragilis^+^ cells aggregated with E12.5 gonadal somatic cells, and 2-cell embryos following *in vitro* maturation (IVM) and fertilization (IVF). (F) Immunofluorescence of SCP1 (red) and SCP3 (green) in aggregates obtained from 6-week old Fragilis^+^ cells with fetal E12.5 gonadal somatic cells 6 days following transplantation. Scale bar = 10 μm. (G–I) Transcriptome of Fragilis^+^ and Fragilis^−^ cells sorted from E12.5 ovaries and Fragilis^+^ cells from 6-week old mouse ovaries. (G) TSNE of global gene expression profiles of Fragilis^+^ cells sorted from fetal ovaries (E12.5 Fra^+^), Fragilis^−^ cells sorted from fetal ovaries (E12.5 Fra^−^) and Fragilis^+^ cells sorted from 6-week ovaries (6W Fra^+^). (H) Scatter plots comparing transcriptome among these three cell populations. Parallel diagonal lines indicate threshold in expression difference. Red, up-regulated genes in E12.5 Fra^+^ cells; blue, down-regulated genes in E12.5 Fra^−^ or in 6W Fra^+^ cells. (I) Heatmap highlighting gene expression profile of germ cells, proliferation and gonad somatic cells

Fragilis^+^ cells isolated from E12.5 or 6-week old ovaries were aggregated with SSEA1^−^ somatic cells sorted from E12.5 ovaries, and subsequently cultured for 24 h and these aggregates looked compact (Fig. S8A). The aggregates of Fragilis^+^ cells sorted from 6-week old ovaries looked similar to those of Fragilis^+^ cells of fetal E12.5 ovaries. Follicles at various developmental stages developed from Fragilis^+^ cells isolated from E12.5 ovaries were readily visible by GFP fluorescence and also in the sections by H&E staining of reconstituted ovaries (Fig. 2C). Moreover, the oocytes expressed both Vasa and GFP and 69 GV oocytes were obtained from three rOvaries. These data indicate that Fragilis^+^ PGCs isolated from E12.5 ovaries can develop into oocytes. To further verify whether Fragilis^−^ cells are able to undergo folliculogenesis and form functional oocytes, Fragilis^−^ cells isolated from GFP mice were aggregated with SSEA1^+^ cells isolated from E12.5 gonad without GFP (Fig. S8A), to reconstitute ovaries. Although follicles and oocytes were developed in the rOvaries, GFP fluorescence was not found in oocytes of the grafts and instead cells with GFP fluorescence were scattered in ovarian stroma (Fig. S8B). These results indicate that SSEA1^+^ cells isolated from E12.5 gonad also can reconstitute ovaries, and further substantiate that E12.5 Fragilis^−^ cells only contribute to somatic cells and granulosa cells, but not oocytes.

Furthermore, we tested the function of oocytes isolated from rOvaries formed from E12.5 Fragilis^+^ cells. These oocytes were able to reach metaphase II (MII), fertilize, and cleave to 2-cell embryos (Figs. S7C–F and 2E). Efficiency of cleavage to 2-cells after in vitro maturation and fertilization was about 71%. Four newborn pups were produced following transfer of 2-cell embryos. The female offspring manifested normal fertility as evidenced by giving birth to live pups after mating with male ICR mice. These data demonstrate that Fragilis^+^ cells from fetal E12.5 ovaries can form fully functional oocytes.

Nevertheless, Fragilis^+^ cells sorted from 6-week old ovaries failed to develop into oocytes in the reconstituted ovary assay (Fig. 2D). Comparatively, the grafts formed from the aggregates of Fragilis^+^ cells sorted from 6-week ovaries were much smaller than the rOvaries derived from fetal E12.5 Fragilis^+^ cells. Follicles were undetectable in the sections of the grafts by GFP and Vasa fluorescence as well as by H&E staining, and correspondingly, oocytes with GFP fluorescence were not found. On the contrary, cells with GFP fluorescence were scattered in ovarian stroma. Therefore, Fragilis^+^ cells from 6-week ovaries fail in oogenesis and follicular development.

To understand the similarities and differences at molecular levels between Fragilis^+^ cells from fetal and postnatal ovaries, or between Fragilis^+^ cells and Fragilis^−^ cells from fetal ovaries, we performed RNA-sequencing (RNA-seq) of the sorted cells. Genome-wide gene expression profile differed remarkably between Fragilis^+^ cells sorted from fetal and postnatal ovaries (Figs. 2G, 2H and S9A), and also between Fragilis^+^ cells and Fragilis^−^ cells from fetal ovaries (Figs. 2G, S9A and S9B). Distinct from Fragilis^−^ cells from fetal ovaries and Fragilis^+^ cells from postnatal ovaries, Fragilis^+^ cells sorted from fetal E12.5 ovaries highly expressed genes representative of PGCs and germ cells, including *Vasa*, *Dazl*, *Blimp1*, *Oct4* and *Tfap2c*, and cell proliferation marker genes *Ki67* and *PCNA*. In contrast, Fragilis^+^ cells sorted from postnatal ovaries only minimally expressed *Oct4* and *Vasa*, although they also expressed *Stella*, validating Western blot and immunofluorescence data shown above. Fragilis^−^ cells however expressed genes associated with gonad somatic cells and granulosa cells, such as *Gata4*, *Foxl2* and *Sox17* (Fig. 2H and 2I). In addition, Fragilis^+^ cells sorted from fetal E12.5 gonad expressed *Fragilis* at much higher levels than did Fragilis^−^ cells, but the expression levels of *Fragilis* did not differ between Fragilis^+^ cells sorted from fetal and postnatal ovaries (Fig. 2H), also supporting the accuracy of MACS sorting by Fragilis.

Moreover, Fragilis^+^ cells from fetal E12.5 ovaries were enriched with genes for homologous recombination, whereas Fragilis^−^ cells enriched with non-homologous end joining (Fig. S9B). Fragilis^+^ cells sorted from fetal ovaries expressed genes involving stem cell maintenance, germ cell development and meiosis and highly expressed genes related to Hippo and mTOR signaling pathways, in contrast to Fragilis^+^ cells of postnatal ovaries (Fig. S9B and S9C). Compared with Fragilis^+^ cells sorted from postnatal ovaries or Fragilis^−^ cells from fetal ovaries, Fragilis^+^ cells from fetal ovaries were highly enriched with genes important for meiosis including *Stra8*, *Spo11*, *Fbxo5*, *Ddx4*, *Sycp1*, *Sycp3*, *Dmc1* and *Rad51* (Fig. S9D and S9E). The transcription profile reveals the molecular insights into differences of Fragilis positive and negative cells. Fragilis^+^ cells from fetal E12.5 gonad are prepared for meiosis entry and progression, whereas Fragilis^+^ cells from postnatal ovaries or Fragilis^−^ cells from fetal E12.5 gonad lack the potential to differentiate into meiocytes.

While germ cells expressing Fragilis only partially express Vasa at E10.5, but with development, Fragilis and Vasa double positive cells increase at E12.5. Expression signal of Fragilis in the gonad begins to weaken at E13.5 and is invisible at E16.5, coincident with meiosis entry and progression. However, Fragilis-marked cells in adult ovaries mostly are distributed in peripheral areas of the follicles and epithelia in the cortex, and do not express Vasa or SSEA1, although Fragilis residue signals can also be found in the oocyte cytoplasm of secondary and antral follicles. Likewise, cytoplasmic expression of Dazl or nuclear expression of Stella is found in Fragilis positive cells at E12.5, but cytoplasmic expression of Dazl or Stella does not co-localize with Fragilis in somatic cells of the postnatal ovaries.

Taken together, Fragilis expression on cell surface, in combination with nuclear Stella or Oct4, does identify oogonia stem cells in embryonic gonads, but in postnatal gonads, it identifies somatic cells rather than oogonia stem cells, demonstrating that this marker protein would not be suitable for isolating putative oogonia stem cells from postnatal ovaries *in situ*. In adult ovaries, Fragilis staining also can be found in the cytoplasm of the oocytes of secondary and antral follicles. Fragilis^+^ cells in postnatal ovaries, despite their unclear originality, mostly are visible in the cortex, stroma and ovarian surface epithelium but are negative for Vasa and Dazl, indicating that these cells are not related to germ cells. Hence, it is not surprising that Fragilis^+^ cells in postnatal ovary could not undergo meiosis in the rOvary. That the same marker can robustly identify oogonia stem cells in the fetal but not in postnatal ovaries further supports the notion that oogonia stem cells could not be readily detected, or are very rare, if any, in postnatal ovaries. Lack of neo-oogenesis in the adult ovaries revealed here by Fragilis is consistent with the finite egg production and follicle reserve not replenishable in adult women and mice. Regardless, the findings that Fragilis can be used as a specific marker for identifying oogonia stem cells may have implications in female reproductive development, infertility treatment and fertility preservation.

## Electronic supplementary material

Below is the link to the electronic supplementary material.

Supplementary material 1 (PDF 1713 kb)
