# Correction to: Characterization of oogonia stem cells in mice by Fragilis

**DOI:** 10.1007/s13238-019-00675-9

**Published:** 2019-11-18

**Authors:** Xiaoyan Sheng, Chenglei Tian, Linlin Liu, Lingling Wang, Xiaoying Ye, Jie Li, Ming Zeng, Lin Liu

**Affiliations:** 1 State Key Laboratory of Medicinal Chemical Biology, College of Life Sciences, Nankai University, Tianjin, 300071 China; 2 Department of Cell Biology and Genetics, College of Life Sciences, Nankai University, Tianjin, 300071 China

## Correction to: Protein Cell 2019, 10(11):825–831 10.1007/s13238-019-00654-0

In the original publication the labelling of Figure 1D, Y-axis is incorrectly published. The correct labeling should be read as Fragilis+/SSEA1+ and the correct Figure 1 is provided in this correction.

**Figure 1:**
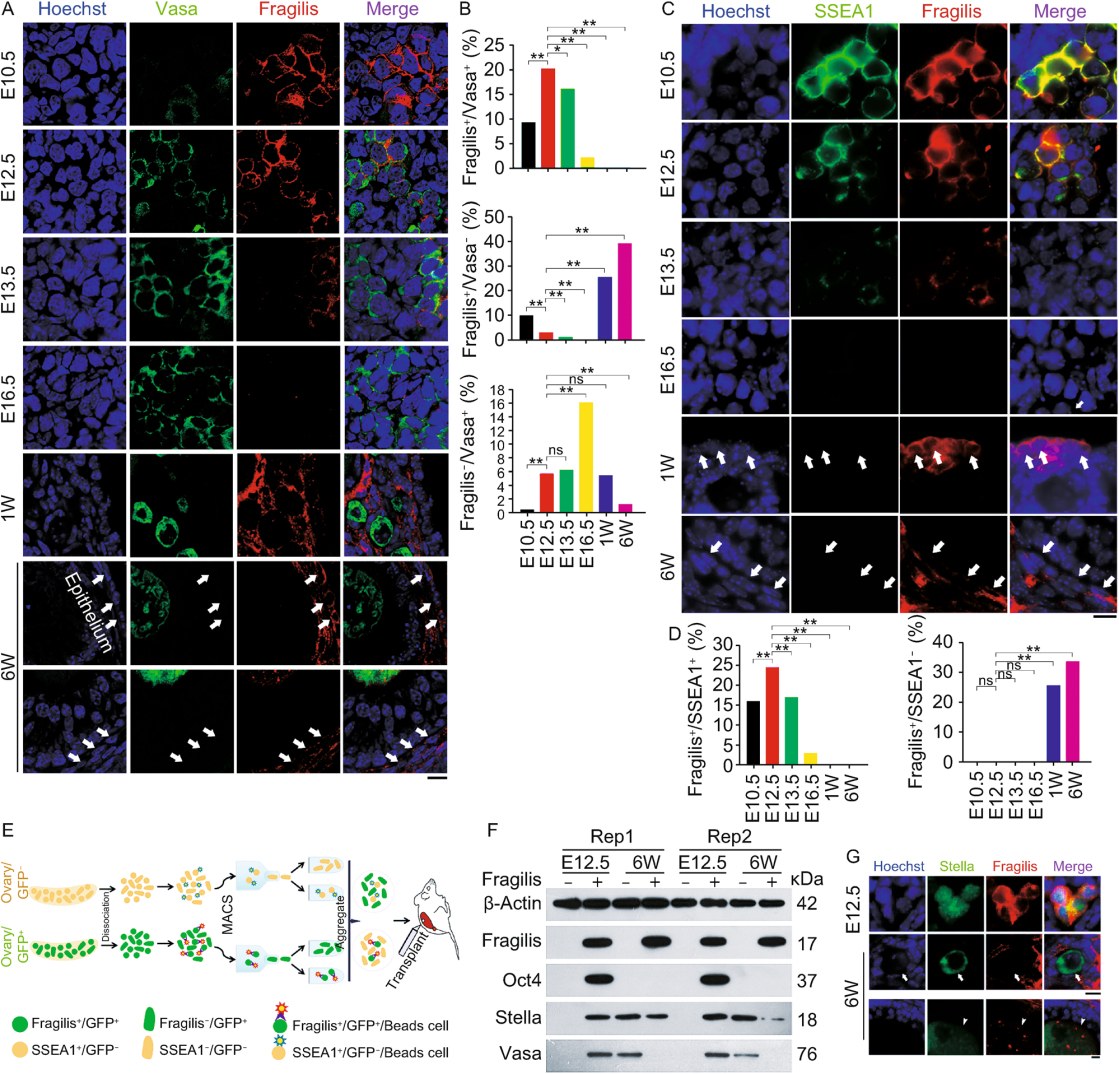
Expression pattern of Fragilis in mouse fetal and postnatal ovaries. (A) Representative confocal images showing co-immunostaining of Vasa (green) with Fragilis (red) in sections of E10.5, E12.5, E13.5 and E16.5 or one week (W) and 6-week old mouse ovaries. White arrows indicate Fragilis^+^/Vasa^−^ cells in the epithelia, stromal or theca cells. Scale bar = 10 μm. (B) Proportion (%) of Fragilis^+^/Vasa^+^ cells, Fragilis^+^/Vasa^−^ cells, and Fragilis^−^/Vasa^+^ cells in mouse ovaries. X^2^ test (*n* ≥ 600 cells counted from three samples for each group). *P < 0.05, **P < 0.01, compared with E12.5 gonad. ns, non-significant difference. (C) Co-immunostaining by epi-fluorescence of SSEA1 (green) with Fragilis (red) in E10.5, E12.5, E13.5 and E16.5, or one week (W) and 6-week old mouse ovaries. White arrows indicate Fragilis^+^/SSEA1^−^ cells in the epithelia, stromal or theca cells. Scale bar = 10 μm. (D) Proportion (%) of Fragilis^+^/SSEA1^+^ cells, and Fragilis^+^/SSEA1^−^ cells in mouse ovaries. X^2^ test (*n* ≥ 600 cells counted). *P < 0.05, **P < 0.01, compared with E12.5 ovaries. Three repeats. (E and F) Isolation and characterization of Fragilis^+^ cells sorted from mouse ovaries. (E) Overview of the protocol for isolation of Fragilis^+^ cells and experimental design for transplantation. (F) Protein expression levels by Western blot of Fragilis, Oct4, Stella and Vasa in Fragilis^+^ cells and Fragilis^−^ cells from E12.5 and 6-week mouse ovaries. β-Actin served as loading control. (G) Co-immunofluorescence of Stella (green) and Fragilis (red) in E12.5 and 6-week old mouse ovaries. Stella mainly in nuclei and Fragilis on the membrane are co-expressed in E12.5 ovaries. In 6-week mouse ovaries, Stella is localized in the cytoplasm of oocytes. Most of Stella positive oocytes are Fragilis negative (white arrows), and few are also positive for Fragilis in which Fragilis is expressed in the cytoplasm of oocytes of secondary and mature/antral follicles (white arrowheads). Scale bar = 10 μm

