# Correction to: Characterization of oogonia stem cells in mice by Fragilis

**DOI:** 10.1007/s13238-020-00743-5

**Published:** 2020-06-25

**Authors:** Xiaoyan Sheng, Chenglei Tian, Linlin Liu, Lingling Wang, Xiaoying Ye, Jie Li, Ming Zeng, Lin Liu

**Affiliations:** 1 State Key Laboratory of Medicinal Chemical Biology, College of Life Sciences, Nankai University, Tianjin, 300071 China; 2 Department of Cell Biology and Genetics, College of Life Sciences, Nankai University, Tianjin, 300071 China

## Correction to: Protein Cell 2019, 10(11):825–831 10.1007/s13238-019-00654-0

In the original publication the labelling on Fig. 2A and B were incorrectly published as E7.5. The correct labelling of Fig. 2A and B should be read as E17.5 which is provided in this correction.

**Figure 2:**
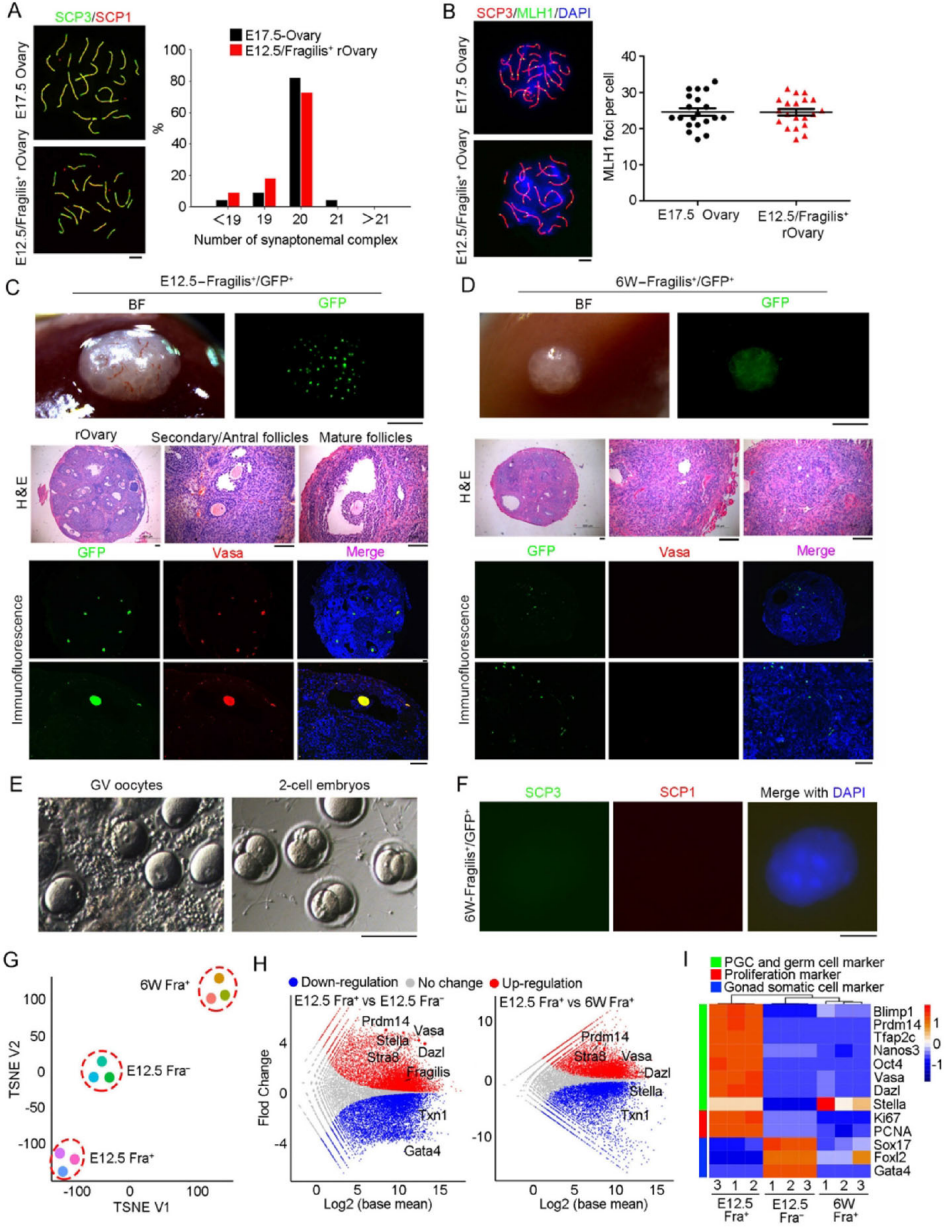
**Neo-meiosis and functional test of oocytes developed from Fragilis**^**+**^ **cells**. (A) Immunofluorescence of SCP1 (red) and SCP3 (green, appear as yellowish by merge with SCP1) showing pachytene spread of E12.5 Fragilis^+^ cells obtained from aggregates with fetal E12.5 somatic cells 6 days following transplantation, compared with those of E17.5 ovaries. Right panel, Percentage of synaptonemal complex elements based on count of spread at pachytene (*n* = 22). Scale bar = 5 μm. (B) Representative immunofluorescence of MLH1 foci at pachytene stage by co-immunostaining of SCP3 (red) and MLH1 (green). Right panel, Statistics of MLH1 foci represents 20 spread per each group. Scale bar = 5 μm. (C) Morphology of reconstituted ovaries 28 days following transplantation of E12.5 Fragilis^+^ cells aggregated with E12.5 gonadal somatic cells into kidney capsules of ovariectomized mice (*n* = 8). Scale bar = 1 mm (upper panel). Middle panel, Follicles are shown in sections by H&E staining. Scale bar = 100 μm. Bottom panel, Co-immunostaining of GFP (green) and Vasa (red) in reconstituted ovaries. BF, bright-field. Nuclei were stained in blue by Hoechst. Scale bar = 100 μm. (D) Morphology of grafts 28 days following kidney capsule transplantation of 6-week Fragilis^+^ cells aggregated with E12.5 gonadal somatic cells (*n* = 3). No follicles but GFP^+^ somatic cells are seen. (E) Morphology of GV oocytes isolated from reconstituted ovaries developed from E12.5 Fragilis^+^ cells aggregated with E12.5 gonadal somatic cells, and 2-cell embryos following *in vitro* maturation (IVM) and fertilization (IVF). (F) Immunofluorescence of SCP1 (red) and SCP3 (green) in aggregates obtained from 6-week old Fragilis^+^ cells with fetal E12.5 gonadal somatic cells 6 days following transplantation. Scale bar = 10 μm. (G–I) Transcriptome of Fragilis^+^ and Fragilis^−^ cells sorted from E12.5 ovaries and Fragilis^+^ cells from 6-week old mouse ovaries. (G) TSNE of global gene expression profiles of Fragilis^+^ cells sorted from fetal ovaries (E12.5 Fra^+^), Fragilis^−^ cells sorted from fetal ovaries (E12.5 Fra^−^) and Fragilis^+^ cells sorted from 6-week ovaries (6W Fra^+^). (H) Scatter plots comparing transcriptome among these three cell populations. Parallel diagonal lines indicate threshold in expression difference. Red, up-regulated genes in E12.5 Fra^+^ cells; blue, down-regulated genes in E12.5 Fra^−^ or in 6W Fra^+^ cells. (I) Heatmap highlighting gene expression profile of germ cells, proliferation and gonad somatic cells

