# Supplementary material for: Characterization of oogonia stem cells in mice by Fragilis
Source: Protein Cell. 2019 Sep 26;10(11):825–31. doi: 10.1007/s13238-019-00654-0 (PMC6834537; doi:10.1007/s13238-019-00654-0)
Supplement: Supplementary file 1 — Supplementary material 1 (PDF 1713 kb) [file 13238_2019_654_MOESM1_ESM.pdf]

Supplementary Information for

Characterization of oogonia stem cells in mice by Fragilis

Xiaoyan Sheng, Chenglei Tian, Linlin Liu, Lingling Wang, Xiaoying Ye, Jie Li, Ming Zeng, and Lin Liu

Materials and Methods

Figs. S1 to S9

Tables S1 to S2

Materials and Methods

Mice and care

Animals care and use of mice for this research were approved by the Nankai University Animal Care and Use Committee. C57BL/6-Tg (CAG-EGFP) C14-Y01-FM131Osb mice that express green fluorescent protein (GFP) in the body were obtained from Model Animal Research Center of Nanjing University. C57BL/6NCrSlc (B6) mice, NOD-SCID mice, albino ICR mice, and albino Kunming (KM) mice were obtained from Beijing Vital River Laboratory Animal Technology Co., Ltd.

Immunofluorescence microscopy of tissue sections

Fetal gonads without attached mesonephros were carefully removed from the female pregnant mice at different growth stages, fixed in 4% paraformaldehyde, dehydrated in sucrose solution and embedded in OCT compound, and sectioned. Sections were immobilized and washed. Sections from paraffin-embedded tissue, after being deparaffinized, rehydration and wash in 0.01 M PBS (pH 7.2-7.4), were subjected to high-pressure antigen recovery sequentially in 0.01 M citrate buffer (pH=6.0) for 3 min.

The sections were permeabilized in 0.1% Triton X-100 for 60 min, incubated with blocking solution (5% goat serum and 0.1% BSA in phosphate-buffered saline, PBS or 3% BSA) for 2 h at room temperature, and then incubated with the primary antibodies overnight at 4°C. Blocking solution without the primary antibody served as negative control. After washing with PBS, sections were incubated with

appropriate secondary antibodies (Alexa Fluor 594 or 488). Nuclei were stained with 0.5 µg/ml Hoechst for 15min, washed, mounted in Vectashield (Vector Laboratories), and photographed with a Zeiss Axio-Imager Z2 Fluorescence Microscope or Zeiss Laser scanning confocal microscope LSM710. The following primary antibodies were used for immunofluorescence: Fragilis (R&D, AF3377), Vasa (ab13840, Abcam), SSEA1 (Millipore, MAB4301), Stella (Abcam, ab19878), PCNA (SC25280, Santa Cruz), Oct3/4 (SC-5279, Santa Cruz), or GFP (AG281, Beyotime Biotechnology). Antibodies were listed in Supplementary Table S1.

For count of immunofluorescence positive or negative cells, three gonads were selected from each mouse group. Maximum sections and two views for each section were selected for each gonad under microscope by the DAPI filter to see just nuclei to avoid of possible bias. The number of cells counted in each group was combined, and at least 600 cells were counted for each group, then relative percentage of positive cells or negative cells also compared. P-value was calculated using X^2 test.

Cell size measurement

Dimensional measurements of different cells were performed using Image J software. Because Fragilis is expressed on the membrane of cell, it is counted by calculating the distance between the membranes.

Magnetic activated cell sorting (MACS)

Isolation and purification of Fragilis positive cells

Ovaries were freshly collected from C57BL/6-GFP mice at the age of E12.5 and 6-weeks. E12.5 ovaries without attached mesonephros were mixed in a 1.5 ml centrifuge tube and dissociated in 0.25% trypsin-EDTA at 37°C for 10 min, neutralized by adding 10% fetal bovine serum (FBS) and dissociated into single cells. Cells from ovaries of 6-week old mice were isolated using the enzymatic digestion method described previously (Zou et al., 2011; Zou et al., 2009), with minor modifications. Briefly, the ovaries were dissected, and cut into a slurry by a sterile ophthalmic scissor. The slurry was incubated at 37°C with intermittent shaking for 15 min in 0.25% trypsin-EDTA and neutralized by adding 10% fetal bovine serum (FBS). Cell suspension from E12.5 ovaries or 6-week mouse ovaries was filtered through a cell strainer with 40-µm pore (Falcon, cat#: 352340), centrifuged, and the supernatant was carefully removed from the pellet. Cell pellet was dissociated into single cells by pipetting in MACS buffer (detailed below) and subject to MACS.

Fragilis positive cells were separated from negative cells by MACS (Woods and Tilly, 2013), according to the manufacturer's instruction (Miltenyi Biotec). Briefly, dissociated gonadal cells were incubated with Fragilis antibody at room temperature for 30 min. After being rinsed and resuspended in MACS buffer (PBS, pH 7.4, added with 0.1% BSA and 2 mM EDTA), the suspension was then centrifuged at 300 g min^{-1} for 5 min to collect the cell precipitate. Cell suspension was incubated with sheep anti-rabbit IgG magnetic beads (Miltenyi Biotec) at room temperature for 20 min, followed by wash in MACS buffer. The mixture of cells and magnetic beads was placed on the magnetic bead separator to obtain Fragilis positive cells. Cell

suspensions dropping from the column were Fragilis negative cells. Fractions on the inner side of the MS column were Fragilis positive cells.

Isolation and purification of SSEA1 positive cells

Ovaries were freshly collected from E12.5 ovaries and dissociated into single cells as described above. MACS was performed according to the manufacturer's instructions (Miltenyi). Briefly, dissociated ovary cells were incubated with anti-SSEA1 antibody conjugated with magnetic beads at 4°C for 20 min. Cell suspensions were washed in PBS supplemented with 0.5% BSA and 2 mM EDTA and applied to an MS column (Miltenyi) to remove SSEA1 positive PGCs. SSEA1 negative cells, also namely gonadal somatic cells, were collected in the flow-through portions.

Aggregates and kidney capsule transplantation

SSEA1 negative somatic cells from fetal E12.5 ovaries were aggregated with Fragilis positive cells from fetal E12.5 ovaries or 6-week old ovaries of C57BL/6-GFP mice, respectively. Also, Fragilis negative cells from E12.5 gonad of C57BL/6-GFP mice were aggregated with SSEA1 positive cells from E12.5 gonad of wild-type mice. These cell aggregates were respectively plated in the wells of a low-cell-binding U-bottom 96-well Lipidure-coat plate and aggregated in MF10 medium (MF10 medium contains M199 (Sigma) with 10% FBS, 1 mM L-Glu, 1% 2A, 50 µg/ml Vitamin C (Vc, Sigma) and 10 µM Rocki), and cultured for 12-18 h at 37°C (Matoba and Ogura, 2011; Zeng et al., 2017). The number of cells in re-aggregated

mass contained 20,000 Fragilis positive cells and 100,000 SSEA1 negative cells, or 100,000 Fragilis negative cells and 20,000 SSEA1 positive cells.

Kidney capsule transplantation was performed based on the methods described (Qing et al., 2008; Zeng et al., 2017). Briefly, one aggregate was picked up with a glass Pasteur pipette and implanted in the “pocket” which was made between the kidney capsule and kidney tissue of a bilaterally ovariectomized female recipient mouse, and 6-8 weeks old NOD-SCID females mice were used as recipients. One aggregate was picked up with a glass Pasteur pipette and implanted in the “pocket”. The transplantation procedure was completed within 5 min for each mouse. Reconstituted ovaries (rOvaries) were obtained and dissected to examine folliculogenesis 28 days following transplantation of the aggregates.

Histological morphology of tissue sections

About 28 days after transplantation, the recipient mice were humanely sacrificed, and the aggregation-formed rOvaries were carefully retrieved from kidney capsule, fixed in 4% paraformaldehyde, dehydrated in gradient ethanol, cleared in xylene, embedded in paraffin, and sectioned for histological examination by hematoxylin and eosin (H&E) staining.

***In vitro* maturation (IVM) and *in vitro* fertilization (IVF)**

The rOvaries were dissected from the recipient mouse kidney capsule, and fully-grown GV oocytes collected in IVM medium under a microscope by pricking follicles using insulin syringe. Oocytes were matured *in vitro* by culture in IVM

medium for 17-18 h at 37 °C. IVM medium contains α-MEM (Invitrogen) added with 5% FBS, 0.24 mM sodium pyruvate (Eppig et al., 2009), 1 IU/ml PMSG and 1.5 IU/ml human chorionic gonadotropin (hCG, Sigma). Oocytes at metaphase II (MII), determined by extrusion of the first polar body, were subjected to IVF.

Spermatozoa were collected from the cauda epididymis of ICR males, capacitated by incubation for 2 h in HTF (Origio), and then incubated with the MII oocytes for 6 h. The zygotes were collected and transferred into human G-1 plus medium (Vitrolife). Embryos that reached the 2-cell stage after 24h culture were transferred into the oviducts of E0.5 pseudo-pregnant mice, and newborns were normally delivered on E19.5. Pups were identified initially by coat color. Contribution of Fragillis positive cells from donor mouse ovaries was confirmed by standard DNA microsatellite genotyping analysis using GFP, D8Mit94 and D12Mit136 primers (Table S2). Pups were mated with albino strain ICR mice to further examine their germline transmission competence.

Immunostaining and fluorescence microscopy of meiocyte spread

Surface spreading of meiocytes was prepared by a drying-down technique (Hodges and Hunt, 2002; Spyropoulos and Moens, 1994) and stained for synaptonemal complex proteins (Liu et al., 2004). Aggregates of Fragilis positive cells from E12.5 ovaries 5~6 days after transplantation and E17.5 ovaries or from adult ovaries were collected, minced with two forceps and dissociated by pipetting in 0.05% TE. After incubation for 7 min at 37 °C, cell suspensions were mixed with an equal volume of FBS, centrifuged for 5 min and resuspended in 100 mM sucrose. Cell suspension

was spread onto glass slide by dipping onto a thin layer of fixative (1% paraformaldehyde, 0.15% Triton X-100 and 3 mM dithiothreitol, pH=9.2). The glass slides were maintained overnight in a humidified box at 4 °C. The slides were washed in water containing 0.4% Photo-flow (Kodak), and completely dried at room temperature. Dried slides were washed with 0.1% Triton X-100/PBS (PBST) for 10min, and incubated with blocking buffer (3% BSA, 2% goat serum/PBST) for 1 h at room temperature. Spreads were then incubated anti-SCP3, SCP1 or MLH1 antibody in blocking buffer at 4°C overnight, washed three times, and then incubated with appropriate secondary antibodies (goat anti-mouse IgG (H+L) FITC or goat anti-rabbit IgG (H+L) AlexaFluor® 594) added with DAPI. The slides were washed and mounted in Vectashield mounting medium (Vector Laboratories). Immunofluorescence was imaged using Axio-Imager Z2 Fluorescence Microscope. MLH1 foci were counted as described (Liu et al., 2004).

Collection of oocytes and immunofluorescence microscopy

GV oocytes were collected from female 6-week old mice 46 h after injection of pregnant mare's serum gonadotrophin (PMSG, 5 IU per mice) by pricking follicles using insulin syringe. Denuded oocytes were fixed and extracted for 30 min at 37 °C in stabilizing buffer. Oocytes were washed extensively and blocked overnight at 4 °C in wash medium (phosphate-buffered saline, supplemented with 0.02% NaN_3, 0.01% Triton X-100, 0.2% non-fat dry milk, 2% goat serum, 2% bovine serum albumin and 0.1 M glycine). Afterwards, oocytes were incubated with Fragilis antibody at 4 °C for overnight or without antibody served as negative control. After washing, samples

were stained for 1:100 actin filaments with Texas Red-conjugated Phalloidin and incubated with appropriate secondary antibodies for 2 h, washed again and counterstained with 0.5 μg/ml DAPI in Vectashield mounting medium (Vector Lab). Fluorescence was detected and imaged using Zeiss Laser scanning confocal microscope LSM710 (Zeiss).

Western blot

Cells were washed twice in PBS, collected, and lysed in cell lysis buffer on ice for 30 min and then sonicated for 1 min at 60 of amplitude at 2 sec intervals. After centrifugation at 10,000 g at 4°C for 10 min, supernatant was transferred into new tubes. Concentration of the protein sample was measured by bicinchoninic acid, and protein samples were boiled in SDS Sample Buffer at 95°C for 10 min. 10 μg total protein of each cell extract was resolved by 10% Acr-Bis SDS-PAGE and transferred to polyvinylidene difluoride membranes (PVDF, Millipore). Nonspecific binding was blocked by incubation in 5% skim milk or 5% BSA in TBST at room temperature for 2 h. Blots were then probed with primary antibodies by incubation overnight at 4°C with Oct4 (sc5279, Santa Cruz), Vasa (ab13840, Abcam), Fragilis (ab15592, Abcam), Stella (ab19878, Abcam) or β-actin (P30002, Abmart). Immunoreactivity bands were then probed for 2 h at room temperature with the appropriate horseradish peroxidase (HRP)-conjugated secondary antibodies, goat anti-Rabbit IgG-HRP (GE Healthcare, NA934V), or goat anti-Mouse IgG (H+L)/HRP (ZSGB-BIO, ZB-2305). Protein bands were detected by Chemiluminescent HRP substrate (Millipore, WBKLS0500).

RNA-sequencing and bioinformatics

RNA-seq libraries were prepared using Smart-seq2 technology as previously described (Picelli et al., 2014). 1000 cells per sample were resuspended in PBS added with 0.1% BSA (A3311-10g, Sigma) and transferred to the bottom of a PCR tube consisting of 3 µl lysis buffer containing oligo (dT) primer and a locked nucleic acid (LNA)-containing template-switching oligonucleotide, and cDNA was synthesized in the tube containing mRNA. Full-length cDNAs were amplified by 18 cycles of PCR using KAPA HotStart ReadyMix (KK2602, KAPA Biosystems). The libraries were prepared by using TruePrep DNA Library Prep Kit V2 for Illumina® (TD503-02, Vazyme Biotech) according to the manual instruction. Samples were barcoded and multiplex sequenced with a 150 bp paired-end sequencing strategy on Illumina Hiseq X10.

Clean reads were mapped to the *Mus musculus* mm10 reference genome using Hisat2 (version 2.1.0). Reads were assigned and counted to genes using Featurecounts (version 1.6.3). The resulting matrix of read counts was then loaded into RStudio (R version 3.5.2), and DESeq2 used to identify differentially expressed genes. Functional enrichment (GO annotation or KEGG) of gene sets with differential expression patterns were performed using clusterProfiler. Heatmaps were drawn by the function “pheatmap” of R packages and correlation coefficients calculation by the function “cor” in R. Scatter plots were generated using the "ggplot2" package to graphically reveal genes that differ significantly between the two samples. P values were adjusted using the Benjamin &

Hochberg method (Hochberg and Benjamini, 1990). Corrected P-value of 0.005 and log2 (fold change) of 1 were set as the threshold for significantly differential expression. Calculated z score of selected genes was used for heatmap.

Statistics

Data were analyzed by X^2 test or fisher's exact test for paired comparison.

Significant differences were defined as *P< 0.05, **P< 0.01 or ***P< 0.001.

Eppig JJ, O'Brien MJ, Wigglesworth K, Nicholson A, Zhang W, King BA (2009) Effect of in vitro maturation of mouse oocytes on the health and lifespan of adult offspring. Hum Reprod 24: 922-928.

Hochberg Y, Benjamini Y (1990) More powerful procedures for multiple significance testing. Stat Med 9: 811-818.

Hodges CA, Hunt PA (2002) Simultaneous analysis of chromosomes and chromosome-associated proteins in mammalian oocytes and embryos. Chromosoma 111: 165-169.

Liu L, Franco S, Spyropoulos B, Moens PB, Blasco MA, Keefe DL (2004) Irregular telomeres impair meiotic synapsis and recombination in mice. Proc Natl Acad Sci U S A 101: 6496-6501.

Matoba S, Ogura A (2011) Generation of functional oocytes and spermatids from fetal primordial germ cells after ectopic transplantation in adult mice. Biol Reprod 84: 631-638.

Picelli S, Faridani OR, Bjorklund AK, Winberg G, Sagasser S, Sandberg R (2014) Full-length RNA-seq from single cells using Smart-seq2. Nat Protoc 9: 171-181.

Qing T, Liu H, Wei W, Ye X, Shen W, Zhang D, Song Z, Yang W, Ding M, Deng H (2008) Mature oocytes derived from purified mouse fetal germ cells. Hum Reprod 23: 54-61.

Spyropoulos B, Moens PB (1994) In situ hybridization of meiotic prophase chromosomes. Methods Mol Biol 33: 131-139.

Woods DC, Tilly JL (2013) Isolation, characterization and propagation of mitotically active germ cells from adult mouse and human ovaries. Nat Protoc 8: 966-988.

Zeng M, Sheng X, Keefe DL, Liu L (2017) Reconstitution of ovarian function following transplantation of primordial germ cells. Sci Rep 7: 1427.

Zou K, Hou L, Sun K, Xie W, Wu J (2011) Improved efficiency of female germline stem cell purification using fragilis-based magnetic bead sorting. Stem Cells Dev 20: 2197-2204.

Zou K, Yuan Z, Yang Z, Luo H, Sun K, Zhou L, Xiang J, Shi L, Yu Q, Zhang Y*, et al.* (2009) Production of offspring from a germline stem cell line derived from neonatal ovaries. Nat Cell Biol 11: 631-636.

Supplementary Figures

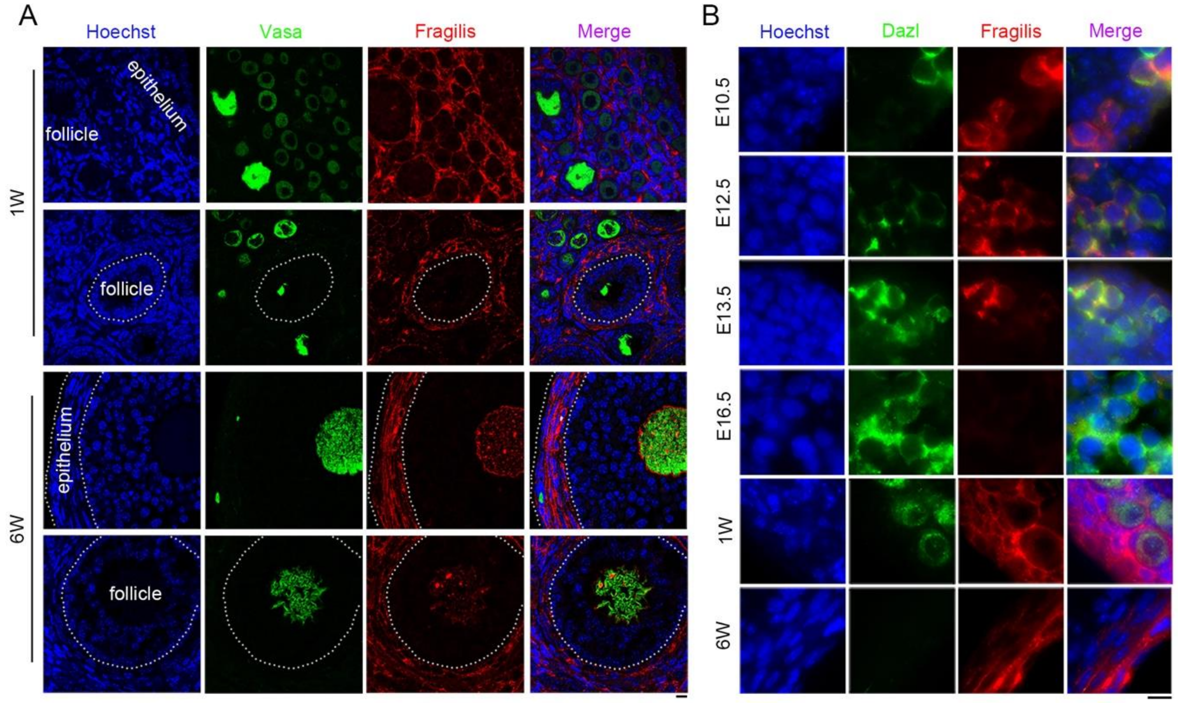

Figure S1. Representative images showing expression of germ cell markers Vasa or Dazl by co-immunostaining with Fragilis in mice ovaries. (*A*) Representative images of Vasa (green) and Fragilis (red) expression in postnatal ovaries from mice at 1-week (1W) or 6-week (6W). (*B*) Co-immunostaining of Dazl (green) with Fragilis (red) in E10.5, E12.5, E13.5, E16.5, 1-week (1W) and 6-week (6W) mouse ovaries. Scale bars = 10 μm.

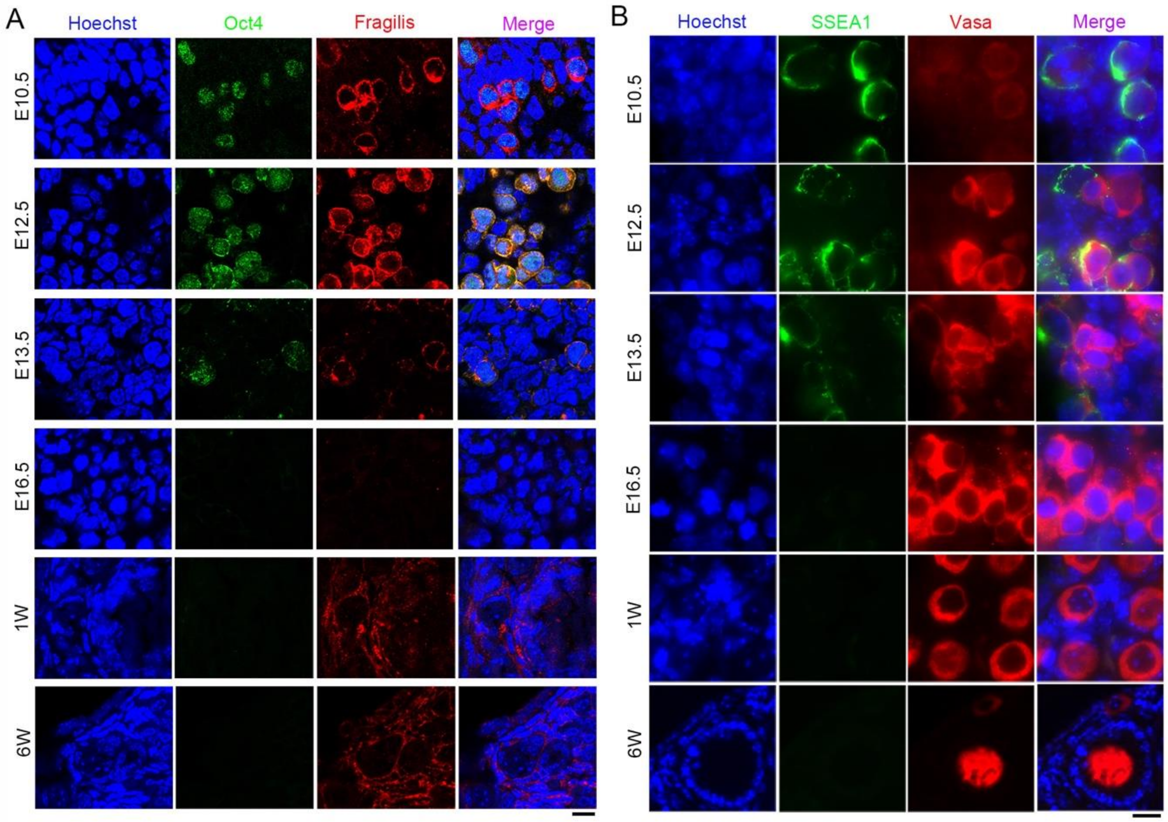

Figure S2. Immunofluorescence of stem cell markers Oct4 or SSEA1 co-stained with germ cell markers Fragilis or Vasa. (*A*) Co-immunofluorescence of Oct4 (green) and Fragilis (red) in E10.5, E12.5, E13.5, E16.5, 1-week (1W) and 6-week (6W) ovaries. (*B*) Co-immunofluorescence of SSEA1 (green) and Vasa (red) in E10.5, E12.5, E13.5, E16.5, 1-week (1W) and 6-week (6W) old mouse ovaries. Scale bars = 10 μm.

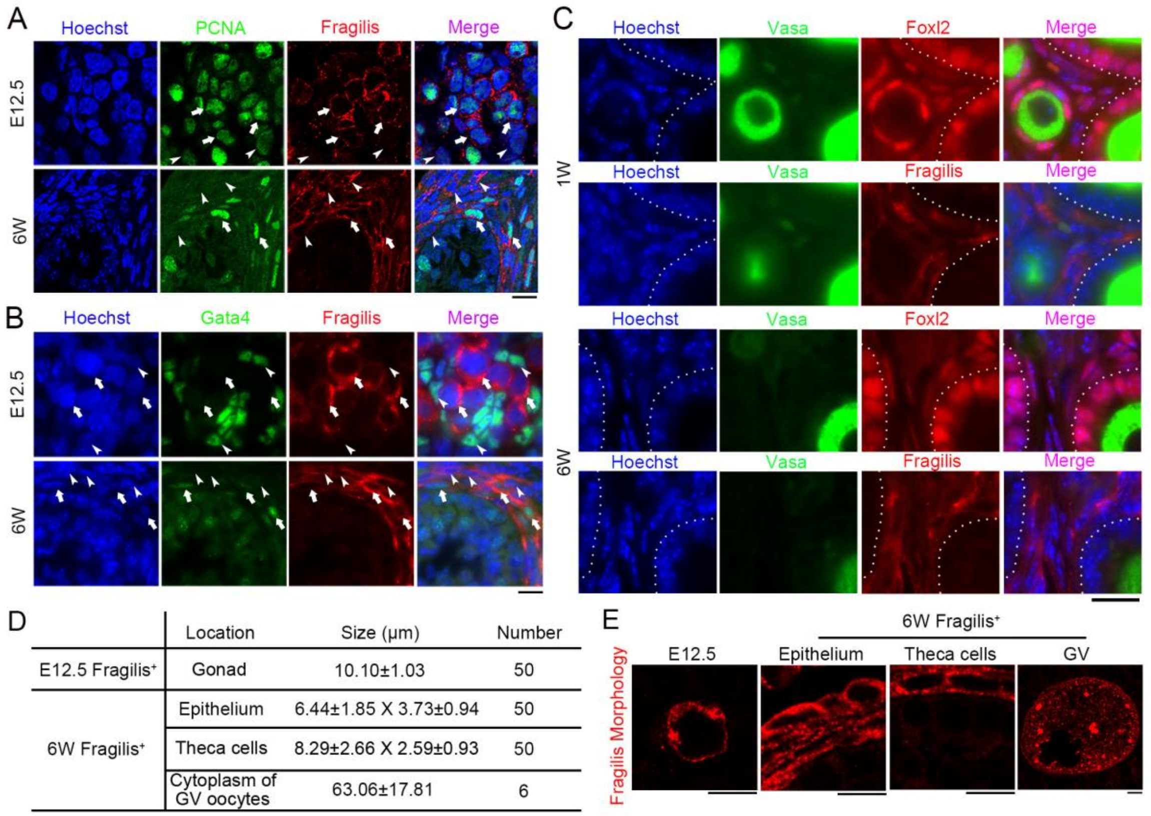

Figure S3. Characterization of Fragilis positive cells in E12.5 and 6-week old mouse ovaries by co-immunostaining *in situ* using various combinations of markers for cell proliferation, granulosa cell and germ cells. (*A*) Co-immunofluorescence of nuclear PCNA and Fragilis. White arrows indicate cells positive for both PCNA and Fragilis. White arrowheads indicate Fragilis negative but PCNA positive cells in E12.5 ovaries or Fragilis positive but PCNA negative cells in 6-week old mouse ovaries. (*B*) Co-immunofluorescence of Gata4 and Fragilis, White arrows indicate Fragilis positive but Gata4 negative cells and white arrowheads indicate Gata4 positive but Fragilis negative cells in E12.5 ovaries. In 6-week old (6W) mouse ovaries, white arrows indicate cells double positive for Fragilis and Gata4. (*C*) Co-immunofluorescence of Vasa with Foxl2, a specific marker for granulosa cells, or Fragilis in the neighbor sections of 6-week old (6W) ovaries. Some Fragilis positive cells can be found to co-localize with theca cells or stromal cells, but not with granulosa cells. (*D*) Comparison of the size of Fragilis positive cells in PGCs of E12.5 and various cell types of 6-week old (6W) ovaries. Number of cells analyzed. (*E*) Morphology of Fragilis positive cells in PGCs of E12.5 and various cell types of 6-week old (6W) ovaries. GV nuclei show residue Fragilis. Scale bars = 10 μm.

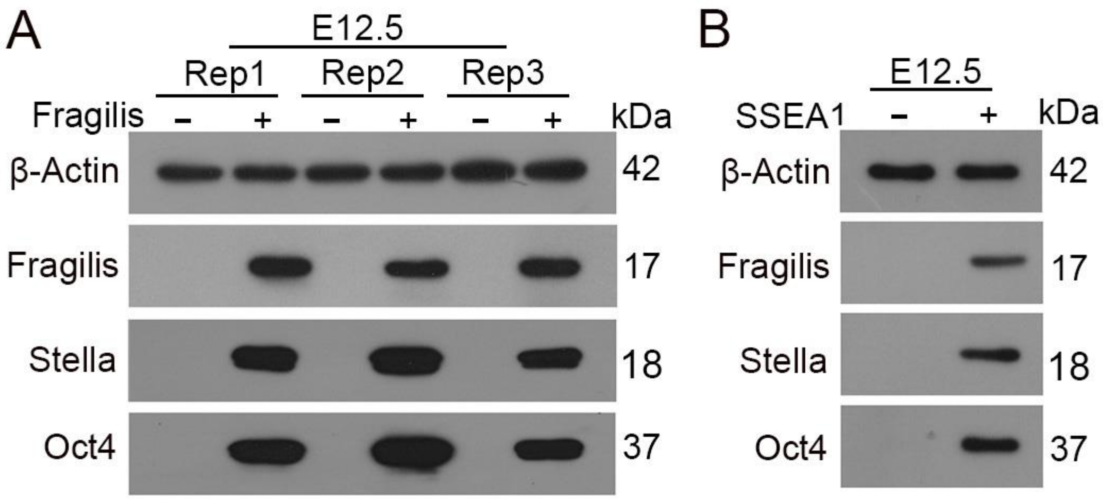

Figure S4. Protein levels by Western blot of Fragilis$^+$ or SSEA1$^+$ cells compared with the negative cells from E12.5 ovaries. (*A*) Protein expression levels of Fragilis, Stella and Oct4 in Fragilis$^+$ and Fragilis$^-$ cells sorted from E12.5 ovaries. (*B*) Protein expression levels of Fragilis, Stella and Oct4 in SSEA1$^+$ and SSEA1$^-$ cells sorted from E12.5 ovaries. β-Actin served as loading control. Rep, replication.

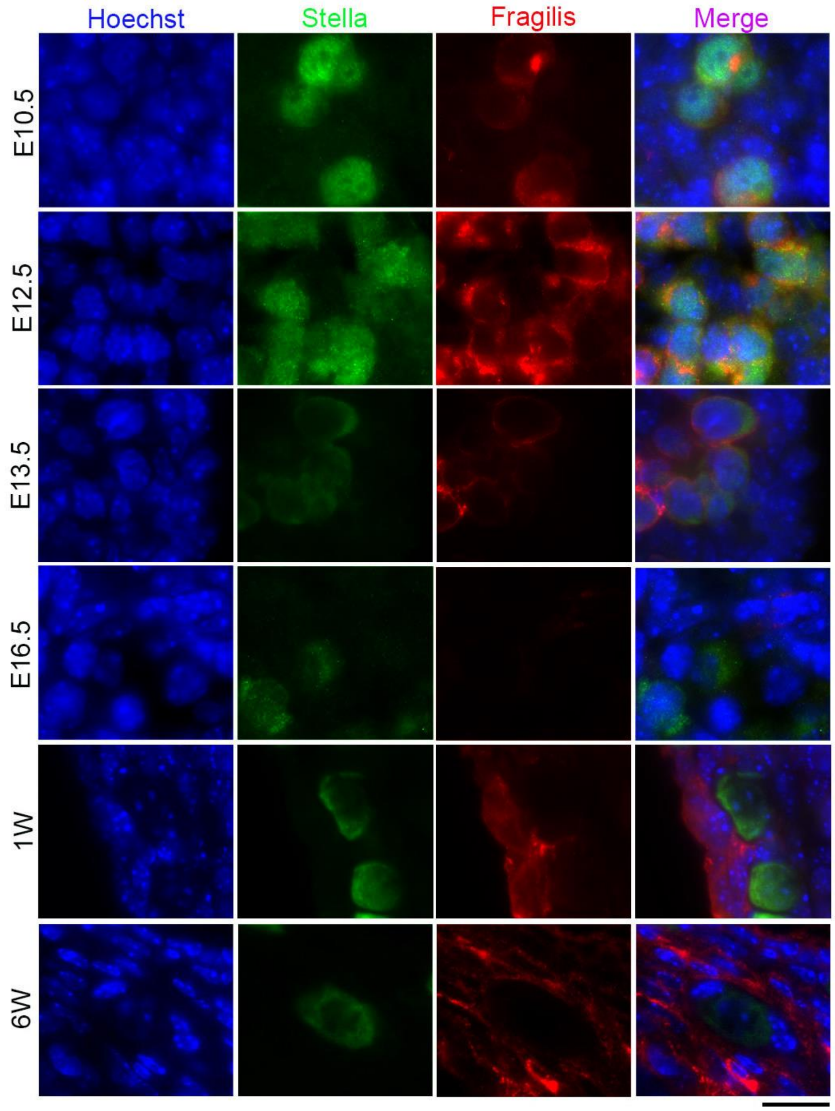

Figure S5. Representative immunofluorescence microscopic images showing expression of Stella (green) and Fragilis (red) in E10.5, E12.5, E13.5, E16.5, 1-week (1W) and 6-week old (6W) mouse ovaries. Note, Stella immunofluorescence is found in the nuclei of PGCs indicated by membrane Fragilis in E10.5 and E12.5 mouse ovaries, but mostly in the cytoplasm of oocytes in 1-week (1W) and 6-week old (6W) mouse ovaries. Scale bar = 10 μm.

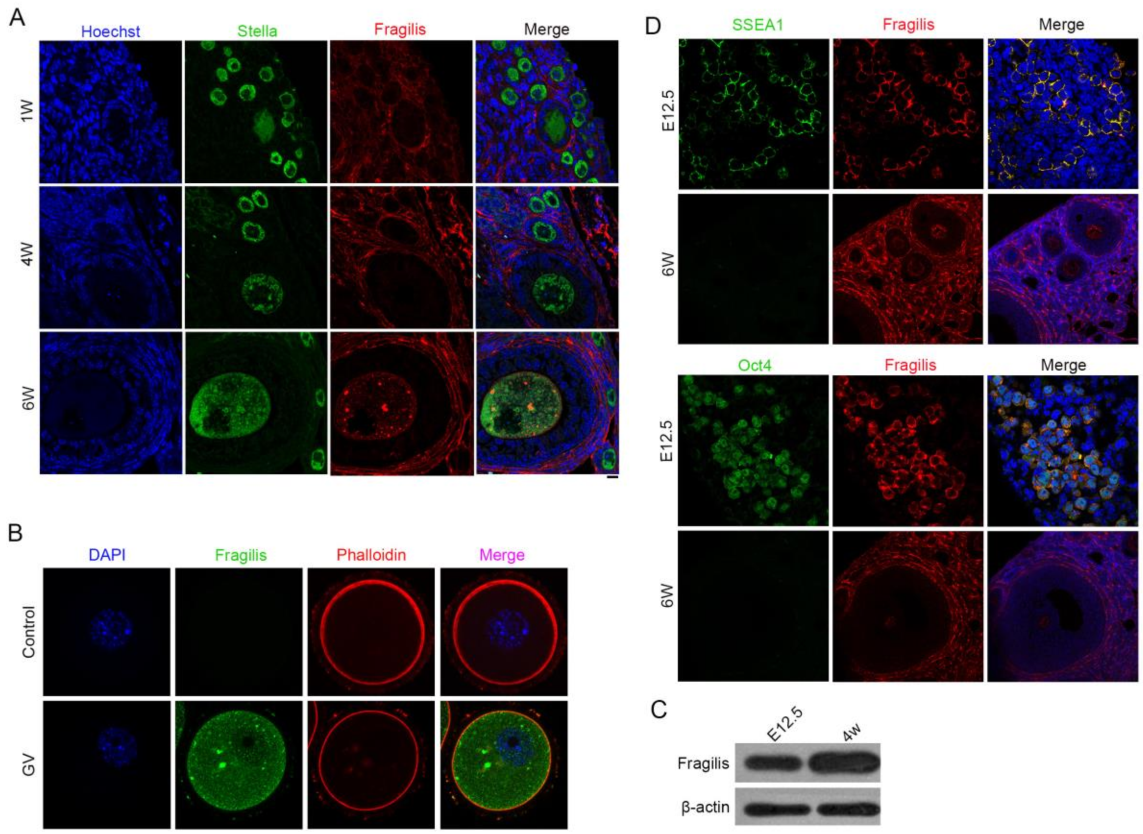

Figure S6. Expression of Fragilis in the oocytes at different developmental stages. (*A*) Representative immunofluorescence confocal images showing expression of Stella (green) and Fragilis (red) in 1-week (1W), 4-week (4W) and 6-week old (6W) mouse ovaries. Hoechst stains nuclei. Scale bar = 10 μm. (*B*) Representative confocal images showing expression of Fragilis in the germinal vesicle (GV) oocytes isolated from 6 week old mouse ovaries. Phalloidin indicates F-actin filament on the membrane. DAPI stains GV nuclei. Control, appropriate secondary antibody is added without primary Fragilis antibody. (*C*) Fragilis protein level by Western blot. Ovaries of 4 to 10-week old mice (shown is 4W mouse) express similar high levels of Fragilis protein, compared with female fetal E12.5 gonad. (*D*) Confocal images of Fragilis with SSEA1 on the surface or Oct4 in the nuclei at lower magnification.

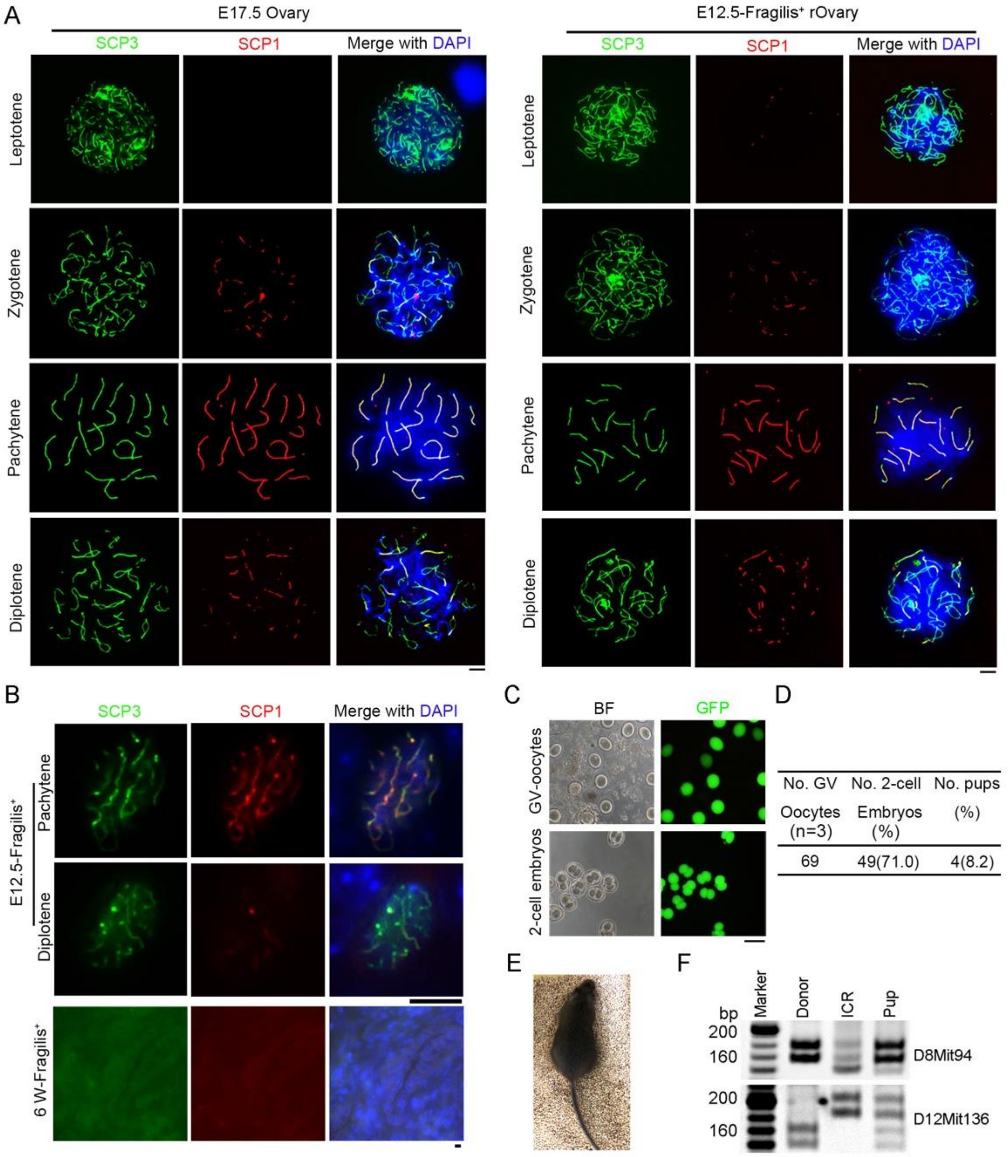

Figure S7. Meiosis of oocytes derived from E12.5 Fragilis$^+$ cells. Immunofluorescence of spread in aggregates formed from E12.5 Fragilis$^+$ cells six days following transplantation and in E17.5 ovaries, revealing SCP1 (red) and SCP3 (green) lateral filaments in meiocytes at leptotene, zygotene pachytene and diplotene stages of prophase I. Scale bar = 5 μm. (*B*) Representative images of pachytene and diplotene meiocytes in sections of the aggregates formed from E12.5 Fragilis$^+$ cells 6 days following transplantation, identified by co-staining of SCP3 and SCP1 (upper), but absence of meiocytes in aggregates formed from Fragilis$^+$ cells of 6-week old (6W) ovaries. Scale bar = 5 μm. (*C*) Morphology and GFP fluorescence of GV oocytes isolated from reconstituted ovaries from E12.5 Fragilis$^+$ cells aggregated with E12.5 somatic cells, and 2-cell embryos following *in vitro* maturation (IVM) and *in vitro* fertilization (IVF). Scale bar = 100 μm. (*D*) Summary of GV oocytes, 2-cell embryos and produced pups following 2-cell embryo transfer. (*E*) Offspring with black-coat produced from embryos of E12.5 Fragilis$^+$ cells-derived oocytes after IVM and IVF with ICR sperm. (*F*) Genotyping of offspring by microsatellite primers D8mit94 and D12Mit136.

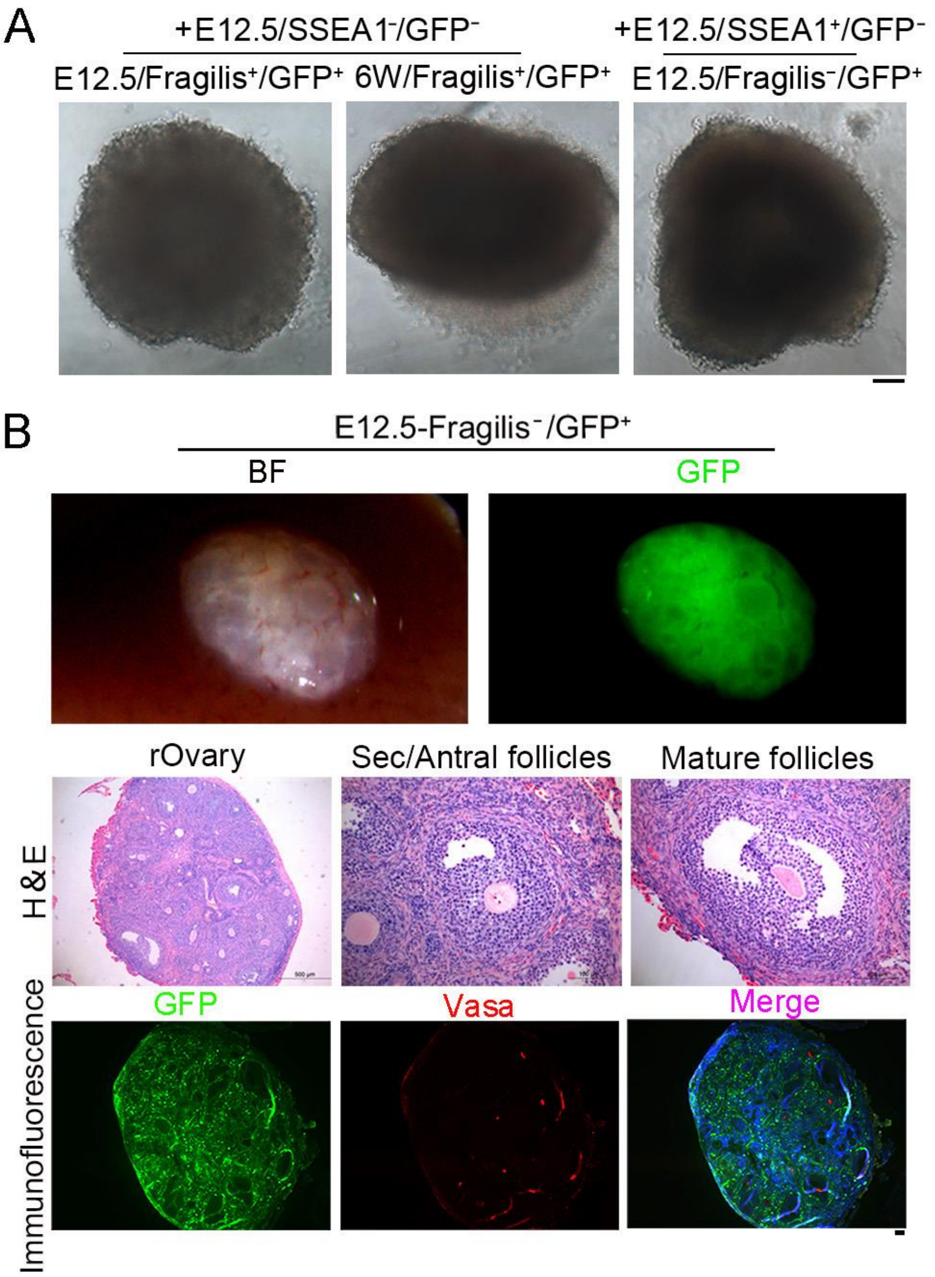

Figure S8. Fragilis⁻ cells from E12.5 ovaries or Fragilis⁺ from 6-week old mouse ovaries do not have the ability to develop into oocytes. (*A*) Morphology of rOvaries after aggregation for 24h in M199+Vc+Rocki. E12.5 Fragilis⁺ cells aggregated with E12.5 SSEA1⁻somatic cells, Fragilis⁺ cells sorted from 6-week old ovaries aggregated with E12.5 SSEA1⁻ somatic cells, or E12.5 Fragilis⁻ cells aggregated with E12.5 SSEA1⁺ cells. Scale bar = 100 μm. (*B*) Morphology of rOvaries 28 days following transplantation into kidney capsules of ovariectomized mice (n = 6) of E12.5 Fragilis⁻ cells from actin-GFP⁺ mice aggregated with E12.5 SSEA1⁺ cells (upper panel). Follicles also are shown in sections by H&E staining (mid-panel). Bottom panel, Co-immunostaining of GFP (green) and Vasa (red) in rOvaries. BF, bright-field. Scale bar=100 μm.

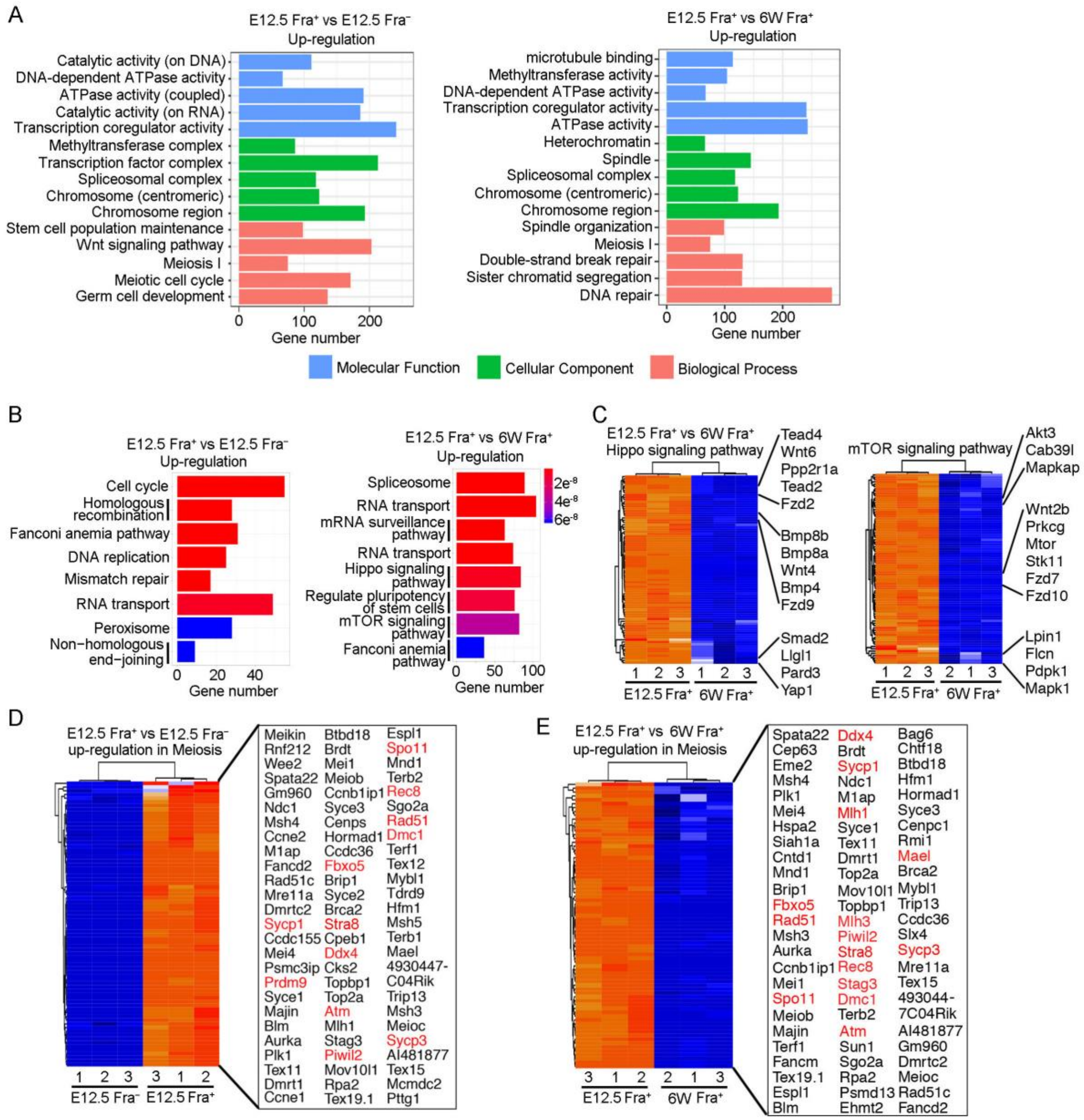

Figure S9. Comparison of transcriptome between E12.5 Fragilis$^+$ cells and E12.5 Fragilis$^-$ cells or 6-week Fragilis$^+$ cells. (*A*) Enriched GO analysis of differentially expressed genes (DEGs) in molecular function, cellular component and biological process between E12.5 fetal Fragilis$^+$ (E12.5 Fra$^+$) cells and E12.5 fetal Fragilis$^-$ (E12.5 Fra$^-$) cells, or between E12.5 Fra$^+$ cells and 6-week Fragilis$^+$ (6W Fra$^+$) cells. (*B*) Enriched KEGG pathways of DEGs between E12.5 Fra$^+$ cells and E12.5 Fra$^-$ cells or between E12.5 Fra$^+$ cells and 6-week Fragilis$^+$ (6W Fra$^+$) cells. (*C*) Up-regulated genes in Hippo signaling pathway (upper panel) and mTOR signaling pathway (bottom panel) in E12.5 Fra$^+$ cells compared with 6W Fra$^+$ cells. Red, white and blue indicates higher, similar and lower relative expression, respectively. (*D*) Up-regulated genes in germ cells and meiosis in E12.5 Fra$^+$ cells compared with E12.5 Fra$^-$ cells. (*E*) Upregulation of genes associated with PGCs and meiosis, homologous pairing and recombination in E12.5 Fra$^+$ cells, compared with those of 6W Fra$^+$ cells.

Table S1. Antibody details

Antibody	Source	Cat. No.
Fragilis	R&D	AF3377
Vasa	Abcam	ab13840
SSEA1	Millipore	MAB4301
Stella	Abcam	ab19878
GFP	Beyotime Biotechnology	AG281
SCP1	Abcam	ab15090
SCP3	Novus	NB300-230
MLH1	BD	550838
Oct4	Santa Cruz	sc5279
PCNA	SC25280	sc25280
β-Actin	Abmart	P30002
Fragilis	Abcam	ab15592
Texas Red-conjugated Phalloidin	Abcam	ab176757
Anti-SCP3- Alexa Fluor® 488 antibody	Abcam	ab205846
Anti-Rabbit lgG Microbeads	Miltenyi Biotec	130-048-602
Anti-SSEA1 conjugated with magnetic beads	Miltenyi Biotec	130-094-530
Goat Anti-Rabbit Alexa Fluor 594	Life technologies	A-11037
Donkey anti-goat Alexa Fluor 594	Life technologies	A-11058
Donkey anti-rabbit Alexa Fluor 488	Life technologies	A-21206
Goat anti-Mouse IgG (H+L) FITC	Jackson	115-095-003
Goat anti-Rabbit IgG-HRP	GE Healthcare	NA934V
Goat anti-Mouse IgG (H+L)/HRP	ZSGB-BIO	ZB2305

Table S2. Primers for microsatellite analysis and GFP analysis

Genes	Forward	Reverse
D8Mit94	GTTGGGGCTCTGCTCTCTC	CACATATGCATACATATACATACACGT
D12Mit136	TTTAATTTTGAGTGGGTTTGGC	TTGCTACATGTACACTGATCTCCA
GFP	TCTCTCCTTAACTACCACCGTCAG	CTGCTTGTCGGCCATGATATAGAC
